# Inflammatory pseudotumor of the liver misdiagnosed as a metastatic tumor of nasopharyngeal carcinoma: a typical case report of mistreatment caused by anchoring bias

**DOI:** 10.3389/fmed.2025.1697002

**Published:** 2025-11-05

**Authors:** Zejin Zhao, Jianli Wang, Yue Xiao, Miaohang Cui, Jian Li, Jing Ma

**Affiliations:** ^1^Department of Hepatobiliary Surgery, The Affiliated Hospital of Chengde Medical University, Chengde, Hebei, China; ^2^Department of Neurology, The Affiliated Hospital of Chengde Medical University, Chengde, Hebei, China

**Keywords:** inflammatory pseudotumor (IPT), misdiagnose, case report, anchoring bias, cognitive bias

## Abstract

This case report describes the clinical process of a patient with a history of nasopharyngeal carcinoma (NPC) who was initially misdiagnosed with a metastatic tumor, later confirmed to be an inflammatory pseudotumor (IPT). The patient was a 68-year-old male individual who was diagnosed with nasopharyngeal carcinoma 4 years ago. The condition was well controlled after regular radiotherapy and chemotherapy. One year ago, a solid mass was found in the left lateral lobe of the liver during routine follow-up. MRI suggested nasopharyngeal carcinoma metastasis. Later, MRI at Peking Union Medical College Hospital also suggested nasopharyngeal carcinoma metastasis. Intrahepatic cholangiocarcinoma was not excluded, and surgical treatment was recommended. Preoperative biopsy was recommended by the multidisciplinary team (MDT); however, the patient declined due to financial constraints and personal preference. The patient underwent laparoscopic left lateral hepatectomy and hilar lymph node dissection at the Affiliated Hospital of Chengde Medical College. Postoperative pathology showed dense infiltration of neutrophils, lymphocytes, and eosinophils, with no malignant components, consistent with an inflammatory pseudotumor. The diagnosis was further confirmed by pathological review and immunohistochemistry at Peking Union Medical College Hospital. The patient recovered well after the operation, and there was no recurrence during 1 year of follow-up. This case suggests that in patients with a history of malignant tumors, even when imaging is highly suspicious of tumors, we should still be vigilant for infectious lesions and avoid anchoring bias. Preoperative biopsy and multidisciplinary comprehensive evaluation (MDT) can help clarify the diagnosis and reduce misdiagnosis and overtreatment.

## Introduction

Liver space-occupying lesions often face the challenge of differential diagnosis in clinical practice, especially in patients with a history of malignant tumors, which are more likely to be misdiagnosed as metastatic lesions or primary liver cancer (1, 2). Inflammatory pseudotumor (IPT) is a kind of benign lesion induced by chronic inflammation, infection, autoimmune reaction, or focal injury. Although it is essentially a non-neoplastic pathological process, it is often manifested as a solid mass in imaging, accompanied by local enhancement, hilar lymph node enlargement, and other “tumor-like” features, which is easily confused with a malignant tumor (3–5). Nasopharyngeal carcinoma (NPC) is a highly prevalent head and neck cancer in China, and the long-term liver metastasis rate can reach 4–15% (6–8). If intrahepatic space-occupying lesions occur during follow-up, metastatic lesions are often considered first (5). This patient was found to have a solid lesion in the left lateral lobe of the liver during the follow-up period after nasopharyngeal carcinoma surgery. Imaging strongly suggested a metastatic tumor. It was misdiagnosed as nasopharyngeal carcinoma, liver metastasis, or intrahepatic cholangiocarcinoma at two top hospitals. Finally, after surgical resection and histopathological evaluation, it was diagnosed as an inflammatory pseudotumor. Although rare, NPC-IPT misdiagnosis has been reported; this case highlights the impact of anchoring bias in a multidisciplinary team (MDT) setting—a context that has been insufficiently explored. This case suggests that in the face of new liver lesions in patients with a history of malignant tumors, clinical diagnosis should not only rely on image characterization but also needs to be combined with pathology, laboratory examination, and comprehensive judgment of the patient‘s overall condition to avoid misdiagnosis and overtreatment due to “anchoring bias.” At the same time, the case also reflects the potential value of preoperative biopsy, EOB-MRI, and other techniques in complex cases, which has important practical significance for improving the ability to identify liver space-occupying lesions and the quality of MDT decision-making.

## Case introduction

**Figure 1:**
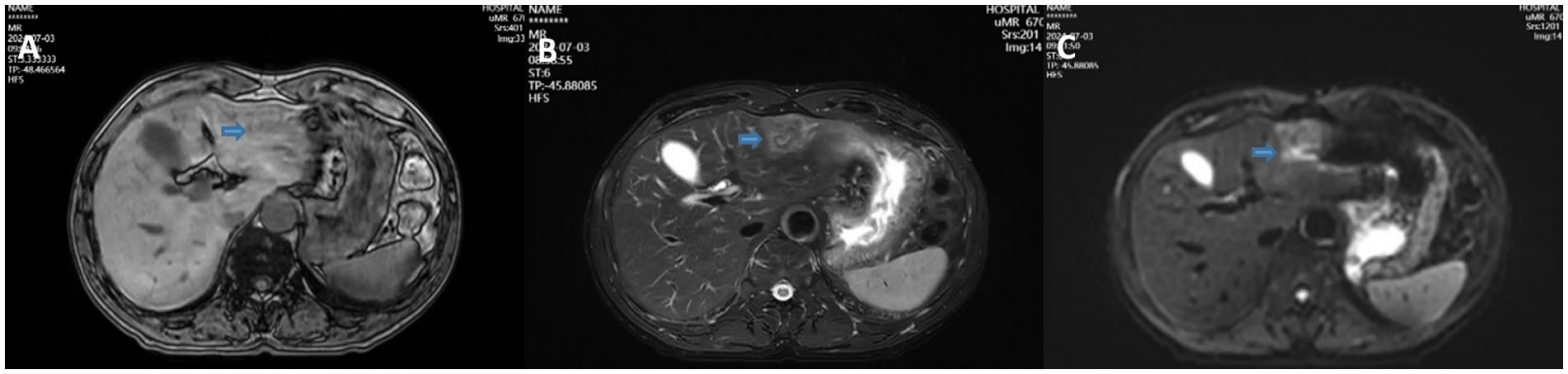
T1WI **(A)**, T2WI **(B)**, and DWI **(C)** showing a solid low-density mass in the left lateral lobe of the liver.

**Figure 2:**
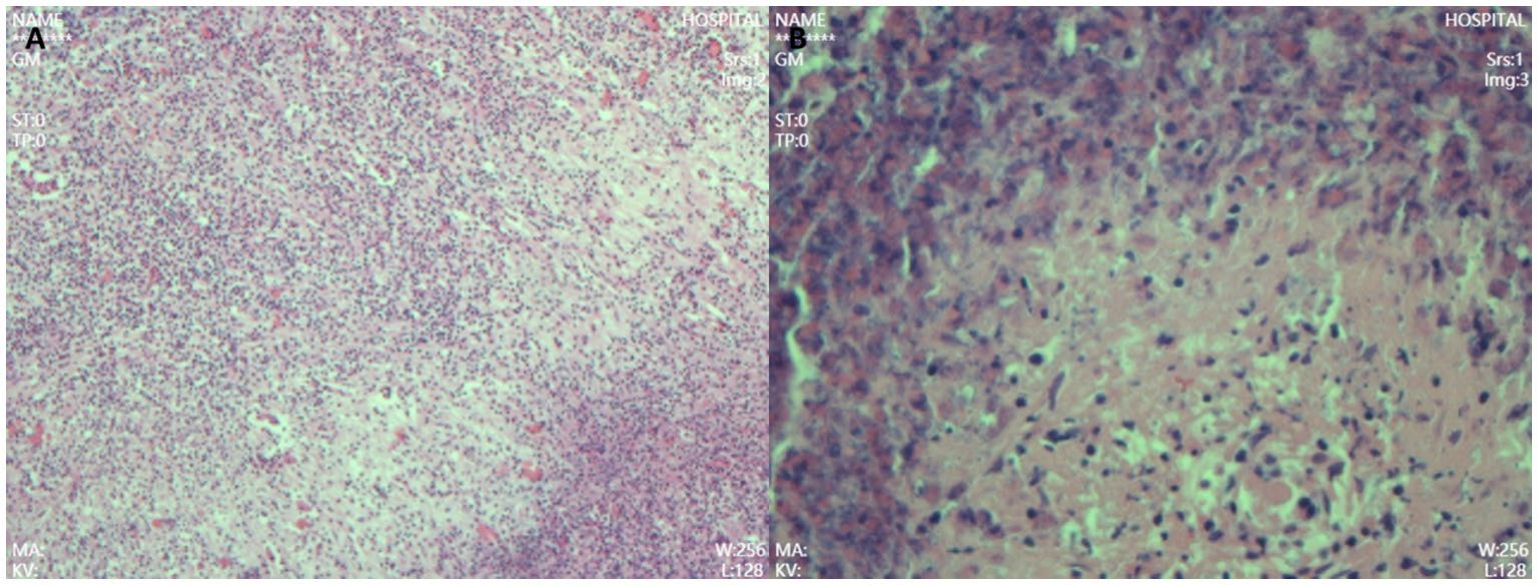
Postoperative pathology showed dense infiltration of neutrophils, lymphocytes, and eosinophils **(A)**, with focal necrosis and granulation tissue hyperplasia but no tumor cells, consistent with an inflammatory pseudotumor **(B)**. (Hematoxylin-eosin staining, high power 200× HE).

A 68-year-old male patient was diagnosed with nasopharyngeal squamous cell carcinoma (T3N2M0, EGFR positive, Ki-67 index 60%) on 20 December 2021, after presenting with headache and nasal congestion. He received concurrent chemoradiotherapy with the GP regimen (gemcitabine plus cisplatin) at the Affiliated Hospital of Chengde Medical College. Post-treatment evaluations performed every 6 months demonstrated no evidence of local recurrence or distant metastasis, and the patient remained clinically stable. On 14 July 2024—approximately 31 months after the initial diagnosis of NPC—routine follow-up CT revealed a solid low-density lesion (5 × 4 × 3 cm) in the left lateral lobe of the liver (T1 hypointense, T2 slightly hyperintense, markedly restricted diffusion on DWI), accompanied by multiple enlarged hilar lymph nodes. The imaging findings were highly suggestive of hepatic metastasis (Figure 1). Serum tumor markers CEA, AFP, and CA19-9 were all within normal ranges. After referral to Peking Union Medical College Hospital, repeat CT/MRI confirmed similar findings and raised suspicion of intrahepatic cholangiocarcinoma. A preoperative liver biopsy was recommended by the multidisciplinary team to clarify the nature of the lesion; however, the patient declined due to financial constraints and personal preference, citing concerns about procedural risk and potential treatment delay. Other diagnostic modalities, such as PET-CT, inflammatory marker testing (CRP, ESR), and gadoxetic acid-enhanced MRI (EOB-MRI), were also not performed for similar economic and logistic reasons. Given the available radiologic and clinical evidence, surgical resection was performed for both diagnostic and therapeutic purposes. The patient underwent laparoscopic left lateral lobectomy and hilar lymph node dissection at the Affiliated Hospital of Chengde Medical College. Intraoperatively, nodular enlargement of the hilar lymph nodes was noted. Gross examination revealed a 14 × 9 × 5 cm specimen of hepatic tissue containing a 5 × 4 × 3 cm nodular mass, along with a 1.5 cm nodular hilar lymph node. Postoperative histopathology demonstrated dense infiltration of neutrophils, lymphocytes, and eosinophils, with focal necrosis and granulation tissue proliferation but no malignant cells, consistent with an inflammatory pseudotumor (Figure 2). The diagnosis was confirmed through histopathological consultation at Peking Union Medical College Hospital. The patient received 2 weeks of postoperative anti-infective therapy, recovered uneventfully, and showed no recurrence at one-year follow-up.

## Discussion

This case was finally diagnosed as an inflammatory pseudotumor (IPT) of the liver, rather than a metastatic tumor, suggesting that IPT has a high risk of misdiagnosis in patients with a history of malignant tumors. Its tumor-like imaging features, non-specific clinical manifestations, and complex and diverse pathological backgrounds together constitute the diagnostic trap. Especially in patients with a history of cancer, clinicians are more susceptible to “anchoring bias”—that is, ignoring new and contradictory evidence due to previous diagnostic impressions (9). Therefore, an in-depth understanding of the pathogenesis, causes of misdiagnosis, and cognitive biases associated with IPT is of great significance for optimizing the diagnosis and treatment path of such cases.

Histologically, IPT is a non-neoplastic benign lesion, usually caused by chronic inflammation, infection, autoimmune response, or local damage. The pathological features are characterized by dense infiltration of inflammatory cells (such as plasma cells, lymphocytes, and macrophages), accompanied by fibroblast proliferation, necrosis, and fibrosis (10). Studies have shown that inflammatory cytokines, such as IL-6, TNF-α, and TGF-β, play a key role in maintaining a chronic inflammatory microenvironment, activating fibroblasts, and inducing local nodule formation (11). The patient had received systemic radiotherapy and chemotherapy. On the one hand, chemotherapy may lead to impaired immune function and reduce the body‘s ability to remove local abnormal inflammatory lesions. On the other hand, radiotherapy-induced liver parenchymal damage and microenvironment disorders are likely to promote chronic repair inflammatory responses. In addition, the effects of radiation and chemotherapy on the intrahepatic biliary structure and liver microcirculation can lead to cholestasis and secondary infection. These changes can further aggravate inflammatory cell infiltration and granulation tissue hyperplasia, thereby forming tumor-like nodules.

Beyond treatment-related injury, recent studies indicate that hepatic IPT encompasses a heterogeneous group of reactive inflammatory lesions with multiple etiologic backgrounds. Approximately one-third of cases are associated with chronic biliary diseases such as cholecystitis, cholangitis, or bile duct obstruction, implying that persistent infection or bile leakage can trigger localized fibroinflammatory reactions within the liver parenchyma (12). Pathogens such as *Escherichia coli*, *Klebsiella pneumoniae*, and *Actinomyces* have been isolated in several reports, supporting an infectious etiology in a subset of patients (13). Another important subset involves IgG4-related disease (IgG4-RD), which is characterized by dense IgG4-positive plasma cell infiltration, storiform fibrosis, and often elevated serum IgG4 levels. IgG4-related hepatic IPTs may coexist with autoimmune pancreatitis or sclerosing cholangitis, and they typically respond well to corticosteroid therapy rather than surgical resection (14, 15). Therefore, immunohistochemical staining for IgG4 and IgG, together with serum IgG4 measurement, is crucial for differential diagnosis. Moreover, trauma, previous surgery, and local ischemia have also been reported as potential triggers of IPT development (16). These findings emphasize that hepatic IPT is not a single pathological entity but a spectrum of reactive fibroinflammatory processes with distinct triggers, ranging from infection and autoimmunity to iatrogenic tissue injury. Understanding this etiologic heterogeneity is essential for accurate diagnosis and individualized management.

In terms of imaging, IPT lacks specific signs and often presents with unclear boundaries, mild-to-moderate T2 hyperintensity, limited diffusion on DWI, and delayed or edge enhancement, making it easily confused with metastases or cholangiocarcinoma (17). In this case, MRI showed high signal on DWI and hilar lymph node enlargement, and the initial impression tended to be metastatic lesions. However, cell density, edema, and necrosis in inflammatory tissues can also lead to increased DWI signals, and hilar lymph node enlargement may also be a reactive change. Although liver-specific contrast agents (such as Gd-EOB-DTPA) can improve the discrimination ability to a certain extent (18, 19), they are not used in this case, which limits the identification of images. In this context, if “contradictory information,” such as asymptomatic patients and normal tumor markers, is ignored, it is easy to form a single path of diagnostic misunderstanding. However, these examinations could have provided additional diagnostic value: PET-CT might have identified occult lesions or assessed metabolic activity suggestive of malignancy; inflammatory markers could have indicated an infectious or inflammatory process; and EOB-MRI, with its hepatocyte-specific contrast, might have helped differentiate inflammatory pseudotumor from malignant hepatic tumors, thereby reducing potential diagnostic bias.

Compared to previous literature, although the misdiagnosis path of this case is representative, it remains relatively rare for IPT to be misdiagnosed as metastasis in the context of a previous malignant tumor history, suggesting that cognitive bias plays an important role in such scenarios. At present, most reports of IPT misdiagnosis are concentrated in populations without a history of malignancy, and diagnostic errors are mainly attributed to the lack of imaging specificity (20). Ke et al. (21) reported a case of an IPT in a patient with hepatitis B infection that was not affected by anchoring bias due to the absence of a prior tumor history. In the present case, the “past cancer history” was repeatedly emphasized as core information during multiple MDT discussions across different institutions, leading to path dependence and a failure to challenge the initial hypothesis of “metastatic tumor.” This phenomenon reflects the amplification effect of medical history as a cognitive anchor. Notably, previous studies have systematically demonstrated that cognitive biases such as anchoring and confirmation bias can strongly influence multidisciplinary diagnostic reasoning, leading teams to prematurely close on an initial diagnosis despite contradictory evidence (22–24). Li et al. (18) summarized the typical MRI features of IPT as delayed enhancement, high DWI signal, and hilar lymphadenopathy, highlighting the potential diagnostic value of EOB-MRI (19). The diagnostic trajectory of this case closely mirrors their findings, further confirming that a “tumor-like image” remains the principal factor contributing to misdiagnosis.

In terms of diagnostic methods, image-guided percutaneous biopsy offers substantial benefits for liver lesions of uncertain etiology. Recent literature demonstrates diagnostic yields as high as 98.9% with minimal delay, comparable to surgical biopsy (25). For intrahepatic cholangiocarcinoma, although an initial non-diagnostic or imaging-pathology discordance rate of 16% has been reported, repeat biopsy resolves many ambiguities (12). The risks of biopsy—bleeding, needle tract seeding, and pain—are real but quantitatively low. Major bleeding events occur in approximately 0.5–1.0% of cases, while minor complications, such as pain or mild hemorrhage, occur in 3–5% (13). Needle tract tumor seeding is relatively uncommon: meta-analyses report an overall incidence of approximately 2.7% in HCC biopsies (or 0.9% per year) and approximately 1% in many large series using modern techniques such as coaxial biopsy (14). Given these data, in cases where imaging findings, clinical history, and biochemical markers are equivocal (as in this case), the balance may favor performing a biopsy, provided that the institution uses proven low-risk techniques (e.g., coaxial needle, minimal passes, and good hemostasis). Abandoning biopsy entirely due to “risk aversion” may lead not only to unnecessary surgical morbidity but also to delayed correct diagnosis and treatment. For example, Lin et al. (15) reported a 32-year-old man whose imaging (CECT, CEMRI, CEUS) strongly suggested intrahepatic cholangiocarcinoma but whose lesion was confirmed as IPT by fine-needle biopsy; the patient avoided surgery. In another Japanese report (16), an IPT of 3.8 cm was observed via US-guided biopsy, and the lesion reduced to 2.6 cm over 3 weeks under observation. Another patient with multiple hepatic IPTs (maximum diameter 33 mm) underwent percutaneous biopsy, was diagnosed with IPT, and had spontaneous regression over 2 months (26). These IPT-specific data suggest that when imaging, tumor markers, and clinical presentation are equivocal, the probability of benign disease is non-trivial; biopsy can meaningfully change management, allowing conservative therapy or surveillance instead of immediate resection.

In addition, this case also reveals the potential limitations of the current MDT mechanism. Although the patient underwent MDT discussions at two tertiary hospitals, the lesion was not recognized as an IPT preoperatively, suggesting that the existing MDT workflow may lack a structured cognitive correction process. The patient’s “history of nasopharyngeal carcinoma” was repeatedly reinforced as an anchoring factor during team discussions, while contradictory evidence—such as normal tumor markers, clinical stability, and absence of immunohistochemical support for malignancy—was underemphasized. Without a structured opposition or evidence-verification mechanism, MDT discussions can inadvertently amplify collective cognitive blind spots, leading to diagnostic or therapeutic misjudgment. To address this issue, future MDT models should integrate structured decision-support tools, such as diagnostic bias checklists, evidence conflict matrices, or “red-team” mechanisms that systematically challenge dominant interpretations. By requiring each participant to explicitly identify uncertainties and opposing evidence before forming a consensus, such tools may help mitigate anchoring and confirmation biases, promote balanced multidisciplinary reasoning, and ultimately improve diagnostic precision and patient safety.

Beyond diagnostic process limitations, this case also highlights the cognitive dimension of diagnostic error, particularly the influence of anchoring bias in clinical reasoning. Anchoring bias, a well-recognized form of cognitive bias in clinical reasoning, refers to the tendency to rely too heavily on an initial piece of information—the “anchor”—when making diagnostic decisions, even in the face of new or contradictory evidence (9). In this case, the patient’s history of nasopharyngeal carcinoma served as a powerful cognitive anchor, which repeatedly guided clinical discussions toward a malignant interpretation of hepatic lesions, despite several discordant findings such as normal tumor markers and the absence of systemic symptoms. This bias can be further reinforced by institutional and multidisciplinary dynamics, where the initial diagnostic label is perpetuated during successive consultations, leading to diagnostic momentum.

To mitigate such bias, several strategies can be implemented at both individual and system levels. First, structured diagnostic reflection should be encouraged during MDT discussions, where clinicians are explicitly prompted to generate and evaluate alternative hypotheses before finalizing decisions. The use of “diagnostic time-outs,” modeled after surgical safety checklists, can help re-evaluate key assumptions and ensure that atypical or contradictory data are given appropriate weight. Second, cognitive debiasing training for clinicians—emphasizing awareness of common biases such as anchoring, confirmation bias, and availability heuristic—has been shown to improve diagnostic accuracy, particularly in complex or high-stakes cases (26). Third, the adoption of decision-support systems integrating clinical, imaging, and laboratory data may provide objective alerts when the diagnostic trajectory is inconsistent with available evidence. Finally, fostering an institutional culture that values diagnostic humility and encourages open challenge among team members can help counteract hierarchical reinforcement of premature conclusions.

In summary, this case underscores that diagnostic excellence not only depends on technological and pathological precision but also on cognitive discipline. Recognizing and actively countering anchoring bias through structured reflection, multidisciplinary transparency, and evidence-based feedback loops are essential to avoid diagnostic errors and ensure patient safety.

## Conclusion

This case is a typical case of an inflammatory pseudotumor misdiagnosed as nasopharyngeal carcinoma metastasis, which exemplifies the risks posed by anchoring bias in the diagnosis of liver space-occupying lesions. Even when imaging features are clear and the clinical presentation is consistent, the awareness of differential diagnosis of infectious lesions should be maintained. Preoperative comprehensive evaluation, image-guided biopsy, and multidisciplinary discussions are the key measures to prevent misdiagnosis. In the future, the integration of advanced imaging methods, such as liver-specific contrast agent MRI, DWI, and PET-CT, is expected to further improve diagnostic accuracy and reduce unnecessary surgical interventions.

## Data Availability

The original contributions presented in the study are included in the article/supplementary material, further inquiries can be directed to the corresponding author.
